# Synthesis, Antimalarial Activity and Molecular Dynamics Studies of Pipecolisporin: A Novel Cyclic Hexapeptide with Potent Therapeutic Potential

**DOI:** 10.3390/molecules30020304

**Published:** 2025-01-14

**Authors:** Nety Kurniaty, Taufik Muhammad Fakih, Rani Maharani, Unang Supratman, Ace Tatang Hidayat, Nurhidanatasha Abu Bakar, Xiaoshuang Wei

**Affiliations:** 1 Department of Pharmacy, Faculty of Mathematics and Natural Sciences, Universitas Islam Bandung, Jl. Ranggagading, Bandung 40116, Indonesia; nety.kurniaty@unisba.ac.id (N.K.); taufikmuhammadf@unisba.ac.id (T.M.F.); 2 Department of Chemistry, Faculty of Mathematics and Natural Sciences, Universitas Padjadjaran, Jl. Raya Bandung Sumedang KM 21, Sumedang 45363, Indonesia; unang.supratman@unpad.ac.id (U.S.); ace.hidayat@unpad.ac.id (A.T.H.); 3 Department of Pharmaceutical Analysis and Medicinal Chemistry, Faculty of Pharmacy, Universitas Padjadjaran, Jl. Raya Bandung Sumedang KM 21, Sumedang 45363, Indonesia; 4 Laboratorium Sentral, Universitas Padjadjaran, Jl. Raya Bandung Sumedang KM 21, Sumedang 45363, Indonesia; 5 Centre of Exploration and Utilization of Natural Resources and Environment Studies, Faculty of Mathematics and Natural Sciences, Universitas Padjadjaran, Jl. Raya Bandung Sumedang KM 21, Sumedang 45363, Indonesia; 6 School of Health Sciences, Health Campus, Universiti Sains Malaysia, Kubang Kerian 16150, Kelantan, Malaysia; natashaa@usm.my (N.A.B.); xiaoshuangwei0@gmail.com (X.W.)

**Keywords:** pipecolisporin, cyclic hexapeptide, solid-phase peptide synthesis (SPPS), antimalarial candidates, computational studies

## Abstract

Malaria, caused by *Plasmodium* species and transmitted by *Anopheles* mosquitoes, continues to pose a significant global health threat. Pipecolisporin, a cyclic hexapeptide isolated from *Nigrospora oryzae*, has emerged as a promising antimalarial candidate due to its potent biological activity and stability. This study explores the synthesis, antimalarial activity, and computational studies of pipecolisporin, aiming to better understand its therapeutic potential. The peptide was successfully synthesized using Fmoc-based solid-phase peptide synthesis (SPPS) followed by cyclization in solution. The purified compound was characterized using HPLC and mass spectrometry, confirming a molecular ion peak at *m*/*z* [M + H]^+^ 692.4131, which matched the calculated mass. Structural verification through ^1^H- and ^13^C-NMR demonstrated strong alignment with the natural product. Pipecolisporin exhibited significant antimalarial activity with an IC_50_ of 26.0 ± 8.49 nM, highlighting its efficacy. In addition to the experimental synthesis, computational studies were conducted to analyze the interaction of pipecolisporin with key malaria-related enzymes, such as dihydrofolate reductase, plasmepsin V, and lactate dehydrogenase. These combined experimental and computational insights into pipecolisporin emphasize the importance of hydrophobic interactions, particularly in membrane penetration and receptor binding, for its antimalarial efficacy. Pipecolisporin represents a promising lead for future antimalarial drug development, with its efficacy, stability, and binding characteristics laying a solid foundation for ongoing research.

## 1. Introduction

Malaria remains a significant global health concern with high mortality rates. It is caused by *Plasmodium*, a parasite transmitted by female *Anopheles* mosquitoes [1]. There are five species of *Plasmodium* that can cause malaria in humans: *Plasmodium falciparum*, *Plasmodium vivax*, *Plasmodium malariae*, *Plasmodium ovale*, and *Plasmodium knowlesi*. Pregnant women, toddlers, and infants are among the most vulnerable groups to malaria fatalities in Indonesia [2]. Regarding malaria treatment, antimalarial drugs that have been widely used over the years include quinine-based drugs and aminoquinoline drugs. One of the commonly used fixed-dose combinations (FDC) is dihydroartemisinin and piperaquine, known as DHP, where each FDC tablet contains 40 mg of dihydroartemisinin and 320 mg of piperaquine. In the quest for alternatives to existing treatments, decades of research have been dedicated to developing antimalarial drugs that are more effective and efficient [3].

**Figure 1:**
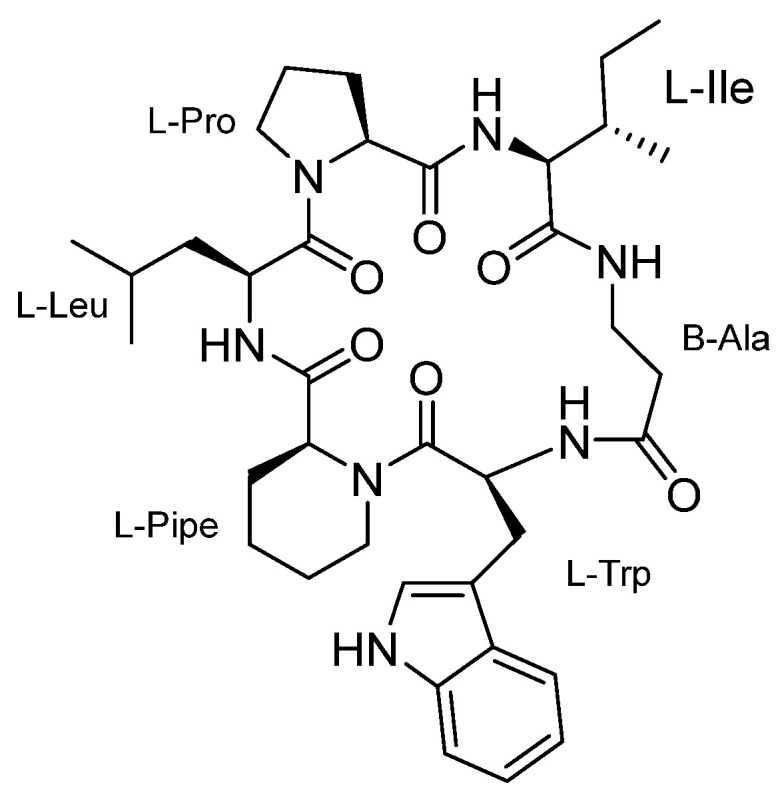
The structure of pipecolisporin.

New drug candidates that have recently attracted attention are a class of peptides, some of which exhibit antimalarial activity [4]. Among these peptides is the ankyrin peptide (AnkP), a potential inhibitor of the *Plasmodium falciparum* cysteine protease falcipain-2, which is delivered to malaria parasite-infected red blood cells (PfRBC) via the homeoprotein internalization domain of antennapedia [5]. In addition to AnkP, synthetic peptides have been reported to specifically target parasite–host or parasite–vector interfaces, such as adhesins involved in host cell invasion [6]. According to a study conducted by Fernández-Pastor et al., 2021 [7], pipecolisporin (Figure 1), which was isolated from the fungus *Nigrospora oryzae*, has demonstrated high antimalarial activity against two different parasite species, *Trypanosoma cruzi* (8.46 μM) and *Plasmodium falciparum* (3.21 μM). This cultivated fungus was isolated from the roots of *Triticum* spp., plants obtained from a traditional plantation located in Montefrio, Granada, Spain.

The cyclic structure of pipecolisporin is considered effective as a drug candidate. Protease enzymes in the body break down amide bonds more slowly in cyclic forms, which means that cyclic peptides have greater binding affinity and exhibit a wide range of biological functions [8,9]. Additionally, cyclic peptides are more efficient at penetrating cell membranes, remain stable within cells, and resist proteolytic degradation. Compared to linear peptides, cyclic peptides are more biologically active and can persist in the human digestive system long enough to reach their intended target [10]. 

To produce pipecolisporin, peptide synthesis is an ideal approach because it allows for the creation of the same compound in a faster, easier, cheaper, and more efficient manner. The chemical synthesis of pipecolisporin has not yet been reported, making it an exciting prospect to explore. Cyclic peptides can be synthesized using a combination of solid-phase and solution-phase methods [11,12,13]. This approach offers a much faster synthesis process compared to using the solution phase alone. Linear peptides are synthesized using the solid-phase approach, while cyclization is accomplished through the solution-phase method. To achieve a good yield of synthetic products, a strategy was developed to synthesize the linear hexapeptide. This strategy involved the careful selection of resin, coupling reagents, protecting groups, and reaction conditions [14].

Computational methods play a crucial role in modern drug discovery, particularly in tackling complex diseases like malaria. Techniques such as molecular modeling, structure-based drug design (SBDD), and high-throughput screening (HTS) accelerate the identification and optimization of potential drug candidates. These approaches allow researchers to simulate interactions between compounds and their targets, like *Plasmodium falciparum*’s key enzymes, providing insights into drug efficacy and potential improvements [15,16]. Machine learning further enhances this process by predicting the biological activity of new compounds, speeding up screening, and reducing costs. In the development of antimalarial peptides such as ankyrin peptides and cyclic compounds like pipecolisporin, computational tools help visualize binding interactions, refine synthesis strategies, and optimize their therapeutic potential. These technologies are invaluable in addressing challenges in drug resistance and improving the efficiency of producing effective antimalarial treatments.

## 2. Results

### 2.1. Synthesis of Pipecolisporin Peptide

**Scheme 1:**
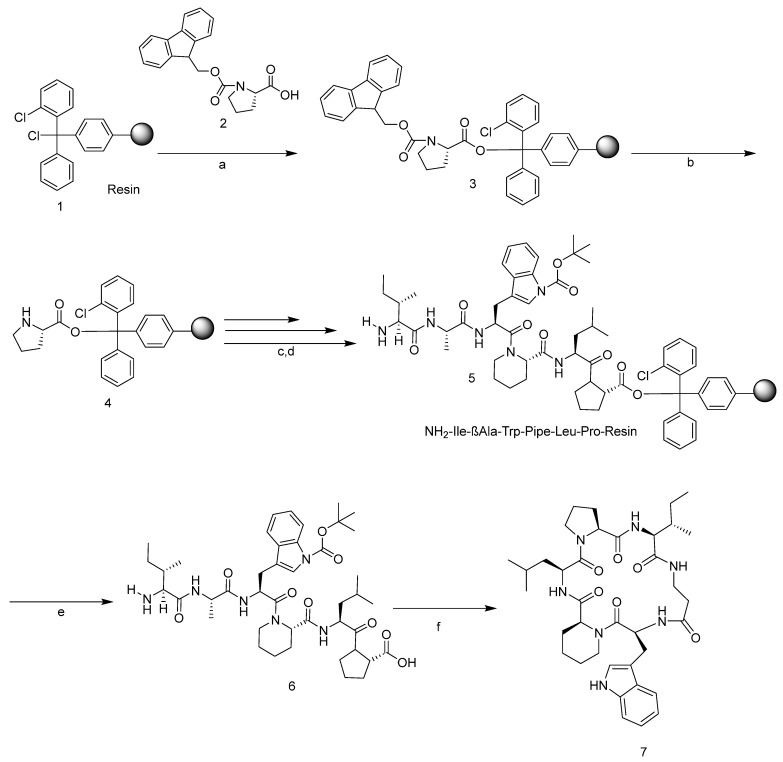
Synthesis of pipecolisporin: (a) (2) Fmoc-Pro-OH, DCM, DIPEA, overnight, (b) (3)Fmoc-Pro-resin, piperidine 20%, DMF, 10 min, (c) (4)NH-Pro-Resin, HBTU (3 ek.), HOBt (3 ek.), Fmoc-Leu-OH (3 ek.), DIPEA (6 ek.) 4 mL DMF, 4 h, RT, (d) Piperidine 20%, DMF, 10 min, (e) TFA in water, 60 min, twice, (f) HATU/HOAt DMF, DIPEA, DCM, 72 h, rt.

Pipecolisporin was successfully synthesized using both solid-phase and solution-phase methods, utilizing the Fmoc strategy on 2-chlorotrityl chloride (2-CTC) resin (Scheme 1). This strategy enables the stepwise addition of amino acids, allowing for precise control over peptide bond formation. The resin loading values obtained were 0.6543 mmol/g for synthetic pipecolisporin. These values fall within the ideal range of 0.2 to 0.8 mmol/g, which is crucial to prevent peptide aggregation during synthesis. If the loading value is too high, aggregation can occur, which negatively impacts the quality of the final product. Conversely, a loading value that is too low may result in reduced yields, making the synthesis inefficient. Therefore, maintaining an optimal resin loading value is essential for obtaining a high-quality peptide product. The gradual addition of amino acids ensures that each step is properly monitored, allowing the final peptide structure to be accurately synthesized.

The coupling of amino acids was achieved using a combination of well-established reagents, including HBTU/HOBt, HATU/HOAt, and DIC/Oxyma, which are known for their high efficiency in forming amide bonds. These reagents facilitate the attachment of amino acids to the growing peptide chain in a manner that minimizes side reactions. Once the amino acids were coupled, linear pipecolisporin was cleaved from the resin using TFA, a standard reagent for cleaving peptides from the resin in solid-phase synthesis. This step is critical because improper cleavage could damage the peptide or leave residual resin attached to the final product. After cleavage, the crude peptide was characterized using mass spectrometry, showing a correct molecular ion peak at *m/z* [M + H]^+^ 810.4730 and, without prior purification, the crude underwent cyclization by stirring the mixture in DCM, DMF, and DIPEA for 72 h at room temperature. Cyclization enhances the stability of the peptide structure, as cyclic peptides are more resistant to enzymatic degradation than linear peptides [8,9,10]. This increased stability makes cyclic peptides more promising candidates for therapeutic applications.

High-resolution time-of-flight mass spectrometry (HR-ToF-MS) was utilized to confirm the successful synthesis of pipecolisporin. This technique is crucial for validating the molecular mass of synthetic compounds, ensuring the absence of unexpected modifications or impurities. For pipecolisporin, the molecular ion peak was detected at *m*/*z* [M + H]^+^ 692.4131 (Figure S3), which closely matched the theoretical calculations, confirming the correct molecular structure had been synthesized. The minor difference of 0.7 ppm indicates a high level of accuracy in the synthesis process. This precision is especially important in peptide synthesis, as even small deviations can significantly impact the compound’s biological activity. With the synthesis confirmed, the next step was to assess the biological activity of pipecolisporin.

### 2.2. Characterization and Purification of Pipecolisporin Peptide

After the synthesis was completed, the crude pipecolisporin peptides were purified using semi-preparative RP-HPLC. The purification process resulted in a single sharp peak for pipecolisporin, with a retention time of 9.992 min (Figure S2), indicating a high degree of purity. This is crucial, as impurities can compromise biological assays and lead to inaccurate conclusions about the compound’s efficacy. Pipecolisporin was obtained with a yield of 16.3 mg (30%) after purification.

Characterization was performed using ^1^H-NMR and ^13^C-NMR at a frequency of 500 MHz with CD_3_OD as the solvent (Table 1, Figures S4 and S5). NMR spectroscopy provides detailed insights into the proton and carbon environments within the peptide, enabling a direct comparison between the synthetic product and its natural counterpart. The NMR spectra for the synthetic pipecolisporin showed a high degree of similarity to those of the natural product, confirming that the synthetic process successfully reproduced the desired molecular structure. There are several signals that are not superimposed between the synthetic and the natural pipecolisporin. This discrepancy is due to the use of different solvents and different frequencies of the NMR instrument used in the measurement. However, confirming the exact number of protons and carbons as specified by the structure of pipecolisporin ensured that we had synthesized the correct molecule. This validation step is critical, as any deviations in structure could potentially impact the peptide’s biological activity. The consistency in the spectra reinforces that the synthesis was accurate, ensuring that the biological function of pipecolisporin is preserved.

### 2.3. Antimalarial Activity Evaluation of Pipecolisporin Peptide

The antimalarial activity of pipecolisporin was evaluated against *Plasmodium falciparum*, the parasite responsible for malaria. Artemisinin, a well-known antimalarial drug, was used as a positive control in this evaluation. The results demonstrated that synthetic pipecolisporin exhibited strong antimalarial activity, with an IC_50_ of 26.0 ± 8.49 nM, which is comparable to that of artemisinin (IC_50_ = 25.5 ± 2.12 nM). This IC_50_ value indicates that pipecolisporin is highly effective at inhibiting the growth of the parasite at very low concentrations, making it a promising candidate for further development as an antimalarial agent. The cyclic structure of pipecolisporin likely plays a key role in its efficacy, as cyclic peptides tend to be more stable and can interact more effectively with biological targets [8,9,10].

The strong antimalarial activity of pipecolisporin, as reflected by its IC_50_ value, suggests that it holds significant potential for further development in malaria treatment. The close similarity in IC_50_ values between pipecolisporin and artemisinin indicates that the synthetic peptide could serve as a viable alternative or complement to existing antimalarial drugs. Pipecolisporin’s cyclic structure likely contributes to its stability and ability to effectively bind to critical targets within *Plasmodium falciparum*, possibly disrupting key processes necessary for the parasite’s survival [17]. Given these promising results, further studies should focus on optimizing pipecolisporin’s structure to enhance its potency and pharmacokinetic properties. Investigating its mechanism of action, potential resistance development, and toxicity profile in comparison to current antimalarial therapies will be crucial steps in advancing pipecolisporin toward clinical application. Additionally, exploring its efficacy in combination with other antimalarial agents could provide insights into whether pipecolisporin might be used synergistically to improve treatment outcomes, particularly in cases where resistance to existing drugs is an issue.

### 2.4. Pipecolisporin Modeling Study

Calculations based on density functional theory (DFT) and the Gaussian interface version 9.0 were utilized to obtain the energy minima structures of pipecolisporin under investigation [18]. The relative energy (Erel) of the optimized geometries of pipecolisporin is −2264.15834444 atomic units (a.u.), which is a measure of energy in quantum mechanics. This value indicates that pipecolisporin possesses very high stability due to the low energy achieved in its optimized geometry. Such stability suggests that pipecolisporin is less likely to undergo spontaneous chemical reactions under normal conditions, as the molecule resides in a minimum energy state, enhancing its overall structural integrity. Additionally, the dipole moment obtained for pipecolisporin is 8.4915 Debye, which reflects the molecule’s ability to interact with external electric fields or other molecules through dipole–dipole interactions. This relatively high dipole moment implies that pipecolisporin may interact strongly with biological receptors or other target molecules, potentially influencing its pharmacological activity. Pipecolisporin also exhibits interactions with various active sites in the molecular environment, with varying binding strengths. This indicates that the compound could have potential applications in scenarios requiring stable binding to specific proteins or enzymes.

### 2.5. Frontiers Molecular Orbital (FMO) Analysis and Reactivity Indicators of Pipecolisporin

A Frontier Molecular Orbital (FMO) assessment was conducted to evaluate the reactivity of pipecolisporin. The differences in energy between the HOMO (Highest Occupied Molecular Orbital) and LUMO (Lowest Unoccupied Molecular Orbital) are crucial for understanding charge transfer behavior [19]. In pipecolisporin, the gap between HOMO and LUMO, measured at 0.18797 a.u., signifies a high level of chemical reactivity. Smaller energy gaps typically indicate higher reactivity, as seen with pipecolisporin when compared to compounds with wider gaps. To deepen the analysis, several reactivity descriptors were determined, including the chemical potential, hardness, softness, and electrophilicity index. The chemical hardness (η) for pipecolisporin, calculated at 0.093985 a.u., points to its enhanced reactivity. Reactivity generally rises as hardness decreases, while stability is inversely proportional to this value. The electrophilicity index (ω), representing pipecolisporin’s electron-accepting capacity, was found to be 0.060233 a.u., further emphasizing its reactive nature over stability. In terms of its electrochemical potential (µ), which reflects electron donation tendencies, pipecolisporin recorded a value of −0.106405 a.u., indicating its ability to act as an electron donor in reactions. Additionally, the nucleophilicity index (N) was measured at 0.106405 a.u., highlighting that pipecolisporin has moderate nucleophilic characteristics, capable of participating in reactions involving electron transfer. These combined metrics indicate that pipecolisporin possesses specific reactivity under certain conditions, allowing it to readily engage in targeted interactions involving electron movement, such as catalytic or binding processes.

### 2.6. Pipecolisporin Structure and Activity Relationship (SAR) Identification

The structure and activity relationship (SAR) analysis was employed to predict the activity of pipecolisporin based on its molecular structure, utilizing the SwissADME tool [20]. Various molecular descriptors, such as the topological polar surface area (TPSA), molecular weight, molar refractivity, and hydrogen bonding capacity, were calculated to assess the physical and chemical properties of the compound. These parameters provide crucial insights into how pipecolisporin might interact with biological targets. The surface area, represented by the TPSA value of 172.81 Å^2^, offers insight into the size and polarity of pipecolisporin. A larger surface area typically correlates with stronger hydrogen bonding interactions with biological targets but may limit membrane permeability. Given its high TPSA, pipecolisporin is likely to exhibit significant interactions with proteins or enzymes, although it may face challenges in crossing cell membranes, which could affect its bioavailability. The molecular weight of pipecolisporin is 691.86 g/mol, which is on the higher side for drug-like molecules. This may also impact its ability to cross biological barriers, such as the blood–brain barrier or cellular membranes, but could support specific, high-affinity binding interactions with target proteins [20]. Hydration energy, which reflects interactions with water molecules, is indirectly suggested by pipecolisporin’s hydrophilicity as indicated by its polar surface area and hydrogen bond donors (5) and acceptors (6). These characteristics suggest a moderate interaction with water, which may support reasonable solubility in aqueous environments, a key factor in bioavailability. The log *p*-value, which is used to measure hydrophobicity, is not provided, but based on its structure, pipecolisporin is expected to have moderate hydrophobic characteristics [21]. This property influences its ability to cross lipid membranes, which is crucial for penetrating cells and exerting its biological effects. In terms of molar refractivity (213.21), pipecolisporin shows a relatively high value, indicating a significant ability to polarize light and interact with its environment, suggesting a higher electron density. This property is often associated with enhanced interactions with biological targets, such as enzymes or receptors, potentially leading to stronger and more effective binding. Overall, these molecular characteristics suggest that pipecolisporin has a good balance between hydrophilic and hydrophobic properties, significant potential for strong interactions with biological targets due to its high refractivity, and reasonable solubility [22]. These features are useful in predicting the compound’s efficacy and potential applications in drug design and molecular modeling.

### 2.7. Molecular Docking Studies

Molecular docking was utilized to estimate the interactions between pipecolisporin and selected protein targets. The docking analysis was performed using the 3D crystal structures of dihydrofolate reductase (2BL9), plasmepsin V (4ZL4), and lactate dehydrogenase (1CET) as biological targets. These proteins were chosen based on their critical roles in the parasite’s metabolism and survival. Dihydrofolate reductase is essential for DNA synthesis and repair, making it a prime target for antifolate drugs; its inhibition disrupts folate metabolism and affects parasite proliferation. Plasmepsin V plays a vital role in exporting parasite-derived proteins into host cells, a process critical for parasite survival and virulence; targeting this protein can interfere with the parasite’s ability to utilize host cell machinery. Lactate dehydrogenase, a key enzyme in the glycolytic pathway, is crucial for energy production; its inhibition can lead to energy depletion, impairing parasite growth and viability. Pipecolisporin was found to interact with the binding pockets of these proteins, forming both hydrophobic and hydrogen bond interactions with key amino acid residues [23]. The binding affinity values for pipecolisporin with the targets are shown in the table, with dihydrofolate reductase exhibiting the strongest binding affinity (−10.26 kcal/mol) and plasmepsin V the weakest (−5.38 kcal/mol). The inhibition constants for these interactions were 29.90 nM and 113.76 µM, respectively, suggesting higher inhibition potential against dihydrofolate reductase (Table 2).

Pipecolisporin’s binding with dihydrofolate reductase involved hydrophobic interactions with PHE57, LEU45, and MET54, along with a hydrogen bond with GLU141. These interactions likely contribute to the compound’s strong binding and inhibition of the enzyme. For plasmepsin V, pipecolisporin interacted with VAL434, LYS432, and LEU179, forming hydrogen bonds with TYR85 and GLU122, but the weaker binding affinity indicates less inhibition potential. Lactate dehydrogenase showed a moderate binding affinity of −6.59 kcal/mol, with key interactions involving residues such as ILE119, PHE100, and LYS118.

**Figure 2:**
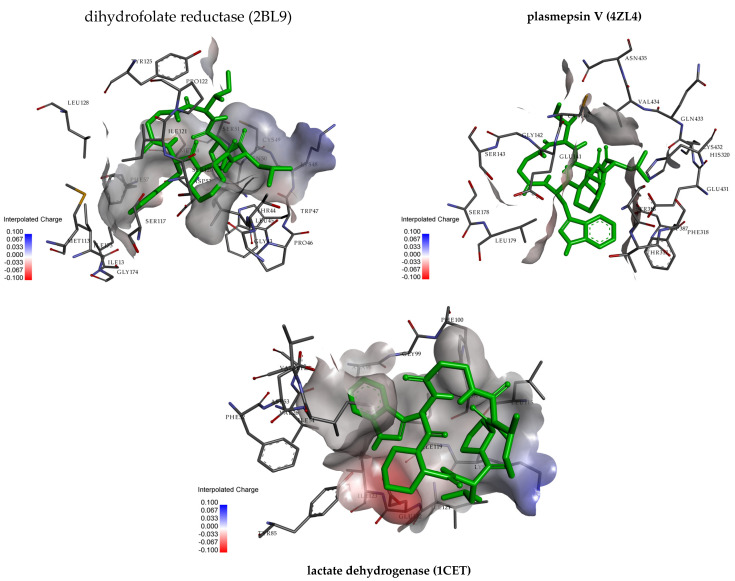
Three-dimensional and residue interactions analysis of pipecolisporin with dihydrofolate reductase (2BL9), plasmepsin V (4ZL4), and lactate dehydrogenase.

The docking results reveal that pipecolisporin can effectively bind to these protein targets, with the strongest interaction seen with dihydrofolate reductase. This suggests that pipecolisporin may have significant inhibitory activity against this enzyme, while its weaker interaction with plasmepsin V implies reduced efficacy. The detailed interactions between pipecolisporin and the binding pockets of the proteins are illustrated in Figure 2, highlighting the role of hydrophobic and hydrogen bond interactions in stabilizing the compound within the active sites of these targets. These findings provide a valuable foundation for further exploration of pipecolisporin as a potential bioactive compound in drug discovery efforts.

### 2.8. Pharmacokinetic and Pharmacodynamics Evaluation

Pharmacokinetics and pharmacodynamics can be used to evaluate the potential of pipecolisporin as a prospective drug candidate. Based on Lipinski’s Rule of Five, the compound was assessed for drug-likeness properties, which are crucial for determining its suitability for further development. For a compound to exhibit drug-like characteristics, it should have a molecular weight of less than 500 Da, fewer than five hydrogen bond donors (HBDs), fewer than ten hydrogen bond acceptors (HBAs), and a log *p*-value of less than 5 [24]. These parameters are used to estimate the compound’s pharmacokinetics, particularly its absorption and distribution potential in the human body. In this case, the lipophilicity (Log *p*) of pipecolisporin was evaluated using various methods, including iLOGP, XLOGP3, WLOGP, MLOGP, and SILICOS-IT, yielding consensus Log *p*-values ranging from −0.14 to 3.78, with an overall consensus Log *p* of 2.01. These values suggest that pipecolisporin has moderate lipophilicity, which indicates a balance between water solubility and membrane permeability, essential for drug absorption. The water solubility of pipecolisporin was also examined, with Log S values (ESOL, Ali, SILICOS-IT) ranging from −5.95 to −8.33, indicating moderate to poor solubility in water. Poor water solubility can limit the bioavailability of the compound, as it affects absorption and distribution within the body [25].

The pharmacokinetic properties of pipecolisporin reveal low gastrointestinal (GI) absorption, poor blood–brain barrier (BBB) permeability, and its classification as a substrate for P-glycoprotein (P-gp), which could influence its distribution and potential drug interactions [26]. Additionally, pipecolisporin was found to inhibit CYP3A4, a critical enzyme in drug metabolism, which may impact its pharmacodynamics and potential drug–drug interactions. Drug-likeness evaluation based on Lipinski’s Rule of Five showed that pipecolisporin had two violations, primarily due to its molecular weight (greater than 500 Da) and number of rotatable bonds. Despite these violations, its bioavailability score of 0.17 suggests moderate absorption potential. The synthetic accessibility score (6.53) indicates that pipecolisporin may be relatively challenging to synthesize, which could impact its development as a drug candidate [27]. Overall, pipecolisporin’s pharmacokinetic and pharmacodynamic profiles suggest that while it possesses certain drug-like properties, its moderate solubility, low GI absorption, and poor BBB permeability could limit its therapeutic potential. Further optimization may be needed to enhance its bioavailability and absorption characteristics. These in silico evaluations provide valuable insights into pipecolisporin’s suitability as a drug candidate and highlight areas for potential improvement in future drug development efforts.

### 2.9. Toxicity Profiles Investigation

Toxicity predictions and biological property evaluations are vital steps in the drug development process. In this case, pipecolisporin was subjected to toxicity and safety analysis using the ProTox tool. ProTox 6.0 was used to predict the potential organ toxicity and other toxicological endpoints, as well as to assess the compound’s safety profile [28]. The results, as shown in the toxicity analysis, predict an LD50 value of 300 mg/kg for pipecolisporin, placing it in Toxicity Class 3, indicating moderate toxicity. The accuracy of the prediction stands at 68.07%, with an average similarity of 64.88%, suggesting reliable predictions. In terms of organ toxicity, pipecolisporin was inactive for hepatotoxicity (85% probability), nephrotoxicity (56% probability), and cardiotoxicity (84% probability). However, it showed active responses for neurotoxicity (63% probability), respiratory toxicity (78% probability), and immunotoxicity (89% probability), indicating potential risks in these areas. It was also active for clinical toxicity (58% probability), which could raise concerns regarding its safety profile for therapeutic use.

### 2.10. Molecular Dynamic Simulations

**Figure 3:**
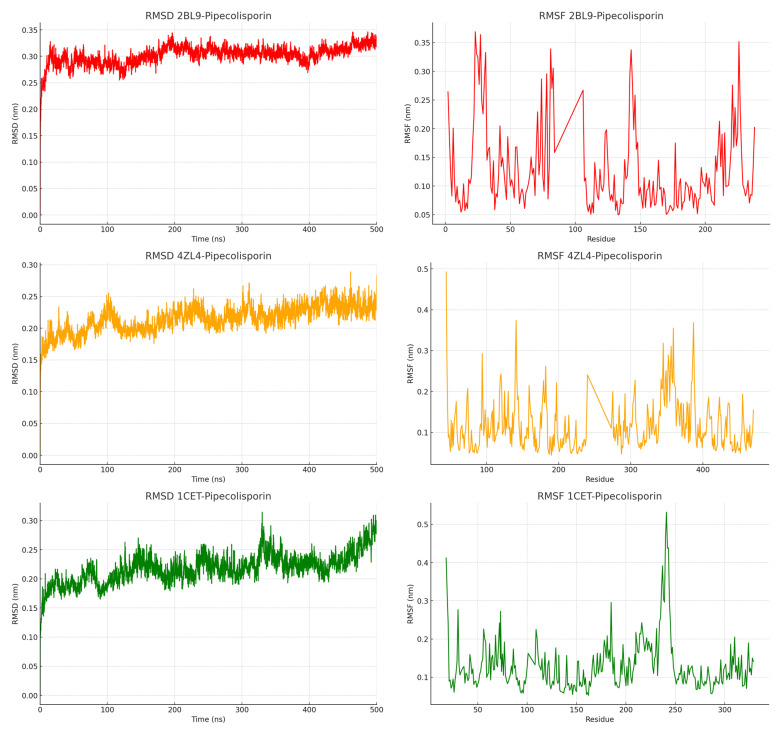
Root-mean-square deviation (RMSD) and root-mean-square fluctuation (RMSF) of the pipecolisporin complex.

Molecular dynamics (MD) simulations were performed on the ligand–protein complexes 2BL9, 4ZL4, and 1CET to examine their structural behavior over a 500 ns timespan. The key analyses included root-mean-square deviation (RMSD), root-mean-square fluctuation (RMSF), Solvent-Accessible Surface Area (SASA), and Radius of Gyration (rGy). RMSD plots for all three complexes demonstrated that after an initial stabilization period of around 50 ns, the proteins maintained stable structures with fluctuations between 0.20 nm and 0.35 nm (Figure 3). This stability indicates that the proteins did not undergo significant structural deviations, suggesting that they are conformationally stable. RMSD for 2BL9 was slightly more volatile early on, but all three complexes reached equilibrium and maintained their structures throughout the simulations. The RMSF plots revealed that certain regions of the protein, particularly in the 2BL9 and 4ZL4 complexes, exhibited higher levels of fluctuation between residues 50 and 150. These fluctuations suggest potential flexible regions in the protein structure, while the rest of the complexes showed lower fluctuations. In comparison, the 1CET complex exhibited smaller RMSF peaks, particularly in the 200–250 residue range, suggesting that 1CET remained structurally more rigid than the other two complexes.

**Figure 4:**
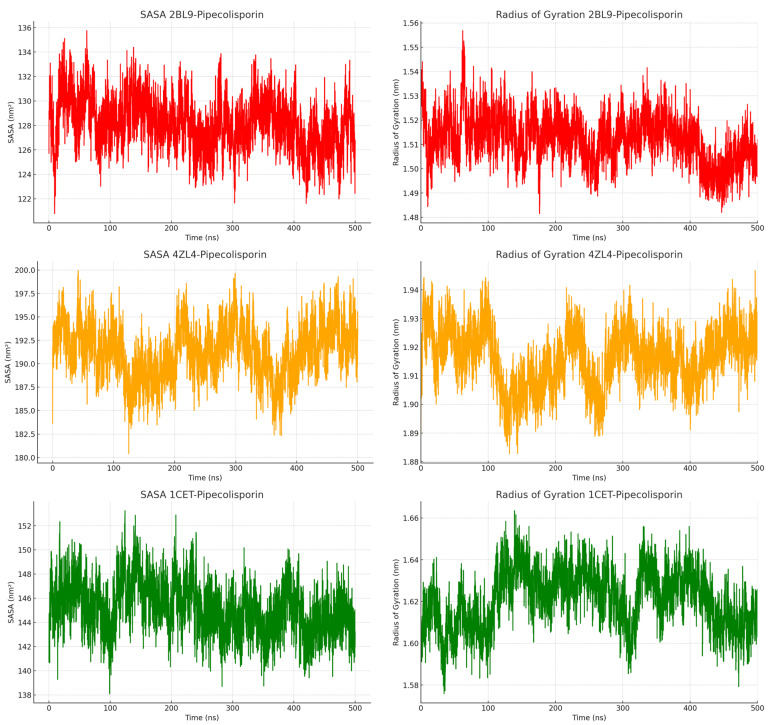
Solvent-Accessible Surface Area (SASA) and Radius of Gyration (rGy) of the pipecolisporin complex.

Based on Figure 4, SASA analysis provided insights into how the proteins’ surface areas interacted with the surrounding solvent throughout the simulation. For the 2BL9 complex, SASA values ranged from 122 nm^2^ to 136 nm^2^, indicating a moderate level of surface exposure to the solvent. The 4ZL4 complex displayed a wider range of SASA values, fluctuating between 180 nm^2^ and 200 nm^2^, suggesting that this complex was more exposed to the solvent environment. The 1CET complex, with SASA values ranging from 138 nm^2^ to 152 nm^2^, displayed relatively less surface exposure than 4ZL4, indicating a more compact structure. Despite these fluctuations, each complex eventually reached an equilibrium state, where the solvent exposure stabilized. The SASA results indicate that the 4ZL4 complex had the most dynamic surface behavior, while 1CET remained more stable and compact. This observation supports the idea that different regions of the protein complexes have varying levels of interaction with the solvent, which can influence the binding behavior of the ligand. The steady-state SASA values suggest that these complexes can maintain their structural integrity while allowing for some flexibility in solvent interactions [29].

The Radius of Gyration (rGy) was calculated for each complex to further assess their compactness and structural integrity during the simulation. For the 2BL9 complex, rGy values fluctuated between 1.48 nm and 1.56 nm, indicating a fairly compact structure with minimal deviations. The 4ZL4 complex exhibited slightly larger rGy values, ranging from 1.88 nm to 1.94 nm, reflecting a more flexible protein structure compared to 2BL9. Similarly, the 1CET complex showed stable rGy values between 1.58 nm and 1.64 nm, maintaining its compact structure throughout the simulation. These stable rGy values indicate that none of the complexes experienced significant expansion or contraction during the simulation period. The results suggest that while some local flexibility exists, the overall structures of these complexes remain intact. Additionally, the consistent rGy values for 1CET suggest that this complex, in particular, may have more rigid structural properties, supporting its reduced fluctuation in both RMSD and RMSF analyses. In summary, the MD simulations show that these ligand–protein complexes maintain stable structures over time, with some flexibility in surface exposure and local movements but no major structural changes, making them suitable candidates for further study in ligand–protein interactions [30].

**Figure 5:**
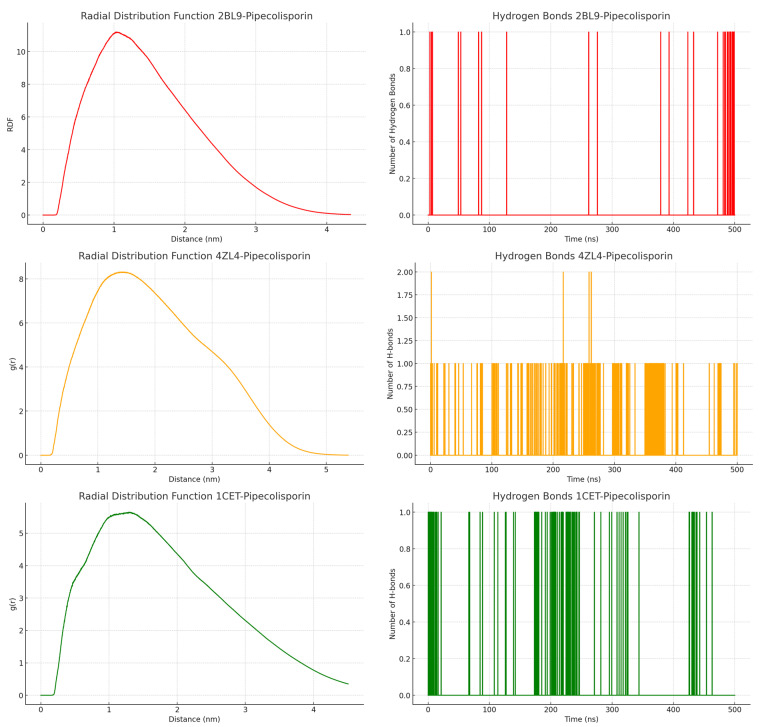
Radial Distribution Function (RDF) and hydrogen bonds analysis of the pipecolisporin complex.

The Radial Distribution Function (RDF) results showed that all complexes had significant ligand–protein interactions at around 1.0 nm, with peaks in RDF indicating strong atomic contacts at this distance (Figure 5). These interactions gradually decreased as the distance increased, signaling fewer contacts beyond 1.5 nm as the systems reached stability. Among the complexes, 4ZL4 showed the highest peak in RDF, suggesting a stronger interaction at the key distance compared to 2BL9 and 1CET. The hydrogen bond analysis further revealed that the 4ZL4 complex consistently formed more hydrogen bonds, fluctuating between 0.5 and 2 bonds, indicating a more stable binding environment. In contrast, 2BL9 and 1CET exhibited fewer hydrogen bonds, with values fluctuating between 0 and 1, implying more transient interactions. These findings suggest that while all complexes exhibit stable ligand–protein interactions, 4ZL4 demonstrates the most stable and consistent binding behavior through stronger hydrogen bonding.

To understand the interaction between pipecolisporin and the target receptors (dihydrofolate reductase, plasmepsin V, and lactate dehydrogenase), binding-free energy calculations were performed using the Molecular Mechanics Poisson–Boltzmann Surface Area (MM-PBSA) method (Table 3). This approach is known for its efficiency in estimating binding energies with reasonable accuracy. The results reveal that pipecolisporin shows notable affinity toward all three target proteins, with binding energy values ranging from −88.392 kJ/mol to −120.386 kJ/mol. The strongest binding was observed with lactate dehydrogenase (1CET) at −94.644 kJ/mol, followed by plasmepsin V (4ZL4) at −88.392 kJ/mol. Dihydrofolate reductase (2BL9) exhibited a binding energy of −120.386 kJ/mol, indicating a strong but slightly less favorable interaction compared to the other targets.

The energy breakdown highlights that van der Waals interactions played a dominant role in stabilizing the ligand within all the complexes, especially with dihydrofolate reductase, where the van der Waals contribution was the largest (−222.699 kJ/mol). Electrostatic interactions were moderate but contributed meaningfully, particularly in the plasmepsin V complex (−28.779 kJ/mol). On the other hand, polar solvation energies opposed binding, with dihydrofolate reductase showing the most unfavorable contribution (150.384 kJ/mol). Solvent-accessible surface area (SASA) energies were negative, reflecting favorable interactions with the solvent. Overall, the data suggest that van der Waals forces are the primary drivers of binding stability for pipecolisporin, while polar solvation poses a slight opposing force.

## 3. Discussion

Pipecolisporin, a cyclic peptide, has shown antimalarial potential that rivals that of artemisinin, which aligns with earlier studies that emphasize the superiority of cyclic peptides in terms of stability and biological efficacy over linear ones. Previous research highlighted its robust antiparasitic action against *Plasmodium falciparum* and *Trypanosoma cruzi*, with IC_50_ values similar to benznidazole, a standard treatment for Chagas disease [7]. Moreover, a comprehensive review of antimalarial peptides pointed out that many peptides isolated or synthesized show significant activity against *Plasmodium falciparum*, and some even inhibit *Plasmodium gallinaceum* and *Plasmodium berghei*. However, some of these peptides also exhibited cytotoxicity toward cell lines like MRC5, HEK2931, and HepG2, which raises concerns about potential toxicity that needs to be addressed in future research [31]. Therefore, although cyclic peptides like pipecolisporin hold promise for their stability and effectiveness in antimalarial treatment, their development should carefully monitor any toxic effects to ensure safe clinical applications.

Research has consistently shown that hydrophobic amino acids like leucine play a crucial role in the bioactivity of peptides [32]. In pipecolisporin, these hydrophobic residues are essential for effective membrane interactions, which are important for the peptide’s biological function. Studies have demonstrated that changes in the composition of hydrophobic amino acids can lead to a reduction in bioactivity. The role of hydrophobicity in maintaining the structural integrity of peptides is key for their ability to interact with cell membranes [32]. This interaction is crucial for many biological processes, including antimicrobial activity and membrane disruption. In the case of pipecolisporin, the presence of hydrophobic residues supports its efficacy in interacting with biological membranes, reinforcing the importance of these residues in peptide function. A study on the antimicrobial peptide Mastoparan-X illustrates that hydrophobic residues promote better membrane integration and are essential for peptide function, while substitutions with polar residues can disrupt these interactions, leading to diminished biological activity [33].

Hydrophobicity is crucial for the ability of peptides to penetrate cell membranes, as it allows the peptide to adopt secondary structures, such as beta-sheets or alpha-helices, upon membrane insertion. These structures are vital for the peptide’s ability to disrupt or translocate across membranes. As shown in other studies, increasing hydrophilicity or reducing hydrophobicity often results in reduced potency, as the balance between hydrophobic and hydrophilic interactions is necessary for maintaining the peptide’s activity against target cells [34]. In the specific case of pipecolisporin, its hydrophobic residues, such as leucine, are essential for the peptide’s ability to interact effectively with the parasitic membrane. Hydrophobicity plays a crucial role in facilitating membrane interactions, allowing the peptide to disrupt or penetrate the membrane. This observation is supported by other research on antimicrobial peptides, where maintaining hydrophobicity is critical for effective membrane-disruptive capabilities. When peptides become more hydrophilic, their ability to disrupt membranes tends to weaken, which in turn reduces their therapeutic potential [35]. In pipecolisporin, the balance between hydrophobic and hydrophilic properties is key to ensuring its effectiveness in biological interactions, particularly in targeting parasitic membranes. Maintaining this balance is crucial for preserving the peptide’s function and therapeutic efficacy.

Based on the computational analysis conducted, pipecolisporin demonstrates promising antimalarial activity, which aligns with previous studies highlighting its potential against *Plasmodium* species. The MM-PBSA calculations further support the stability and effectiveness of pipecolisporin in binding to key target receptors like dihydrofolate reductase, plasmepsin V, and lactate dehydrogenase. This finding corroborates earlier research that emphasizes the superior stability and biological efficacy of cyclic peptides like pipecolisporin compared to linear peptides [36]. Interestingly, the study also touches on the critical role of hydrophobic interactions, particularly van der Waals forces, in stabilizing the ligand–protein interactions. Furthermore, concerns about potential cytotoxicity, as observed in related peptides, indicate that future studies on pipecolisporin should carefully assess its safety profile to mitigate any harmful effects while enhancing its therapeutic potential.

## 4. Materials and Methods

### 4.1. Tools and Materials

The tools that we used in this study are glassware commonly used in synthesis laboratories and other supporting tools such as SPPS reactor (C.V Rizky Glassindo, Cileunyi, Indonesia), heating mantle (Thermo Scientific, Waltham, MA, USA), rotatory evaporator (Buchi, Flawil, Switzerland), freeze dryer (Ihanil Vac 8, Gimpo, Republic of Korea), and balance (Mettler Toledo, Columbus, OH, USA). Monitoring of cyclization was carried out by thin-layer chromatography (TLC) of silica gel GF254 (Merck, Darmstadt, Germany) with λ 254 and 365 nm UV detector lamps (C.V Ruchi, Bandung, Indonesia). Characterization of pure compounds used mass spectroscopy with HR-TOF-MS (High-Resolution Time-Of-Flight Mass Spectroscopy) from Lockspray Waters (Milford, MA, USA), Proton-Nuclear Magnetic Resonance (^1^H-NMR), and Carbon-Nuclear Magnetic Resonance (^13^C-NMR) using Bruker Ascend Spectrometer (Billerica, MA, USA) at 700 MHz for ^1^H, 125 MHz for ^13^C and TMS as an internal standard. Compound purity analysis was performed with analytical RP-HPLC using Water Alliance 2998 PDA (Photodiodearray) detector with C-18 Acquity BEH column 1.7 μm (2.1 × 50 mm), and purification used semi-preparative RP-HPLC using Water Alliance e2695 PDA (Photodiodearray) detector 2489 with RP-18e LichroCart column 5 μm (4.6 × 250 mm), microplate reader (Molecular Devices LLC, San Jose, CA, USA), probit regression analysis (GraphPad Prism software, Version 8, San Diego, CA, USA).

The chemicals used in this study were amino acids Fmoc-L-Pro-OH, Fmoc-L-Leu-OH, Fmoc-L-Pip-OH, Fmoc-L-Trp(Boc)-OH, Fmoc-L-β-Ala-OH, Fmoc-L-Ala-OH, Fmoc-L-Ile-OH, Hexafluorophosphate aza-benzotriazole tetramethyl uranium (HATU), Hexafluorophosphate benzotriazole tetramethyl uranium (HBTU), 1-hydroxy-7-azabenzotriazole (HOAt), 1-hydroxybenzotriazole (HOBt), *N,N’*-diisopropylcarbodiimide, (DIC), ethyl cyanohydroxyaminoacetate (OXYMA), 2-chlorotriethylchloride (CTC) resin (GLBiochem, Shanghai, China), acetaldehyde, *N*,*N*-diisopropylethylamine (DIPEA), dimethylaminopyridine (DMAP), glacial acetic acid, dichloromethane (DCM), dimethylformamide (DMF), methanol, ethyl acetate, *n*-hexane, piperidine (trifluoroethanol (TFE), trifluoroacetic a cid (TFA), dimethylsulfoxide (DMSO), acetonitrile grade HPLC, methanol grade HPLC, plate KLT silica gel GF254, Nutrients Agar (NA), Muller Hinton Agar (MHA), Muller Hinton Broth (MHB) (Merck, Darmstadt, Germany), *N*,*N*-diisopropylcarbodiimide (DIC), *p*-chloranyl (Sigma Aldrich, Darmstadt, Germany), aquades, chloramphenicol, cultures of *Plasmodium falciparum* and *Trypanosoma cruzi*, D-sorbital (Sigma-Aldrich, St. Louis, MO, USA), Artemisinin (Sigma-Aldrich, St. Louis, MO, USA), untreated parasites and complete culture medium (RPMI, Gibco Laboratories, Norristown, PA, USA), GelGreen^®^ (Sigma-Aldrich, St. Louis, MO, USA).

### 4.2. Synthesis of Pipecolisporin Linear Peptide

The 2-chlorotritylchloride resin (capacity 0.99 mmol/g, 250 mg) was placed in the reactor. Subsequently, 5 mL of DCM was added, and the reaction mixture was shaken for 15 min, followed by filtration and drying of the resin. The dried resin was then combined with a solution of Fmoc-L-Pro-OH (0.375 mmol) in 3 mL of DCM, along with DIPEA (0.75 mmol). This mixture was shaken overnight at room temperature. After completing the reaction, the resin was filtered, washed with DMF and DCM, and dried. The dried resin was capped with 5 mL of methanol:DCM (2:7:1) and shaken for 2 × 10 min. Subsequently, the resin was rinsed with DMF and DCM and dried. Several resin grains were taken to determine resin loading. The dried resin-Pro-Fmoc was treated with a solution of 20% piperidine in DMF (4 mL). The reaction mixture was shaken for 2 × 5 min, filtered, washed with DMF and DCM, and dried. The dried resin was tested with a chloranil solution by applying three drops of 2% p-chloranil in DMF and three drops of 2% acetaldehyde in DMF onto a small amount of resin in a microtube. The color change of the resin from yellow to blue or green indicated the successful formation of free amines and deprotection. The first amino acid-bound resin (resin-Pro-OH) was treated with a solution containing the corresponding amino acid, coupling reagent, and DIPEA in a 3:3:6 ratio. The reaction mixture was shaken for 4 h at room temperature. After completion of the reaction, the resin was filtered, washed with DMF and DCM, dried, and tested using a chloranil solution. The dried resin was then deprotected from Fmoc. HBTU/HOBt reagents were used as coupling agents in most amide bond formations, except for Fmoc-Trp(Boc)-OH coupling, which utilized a combination of HATU/HOAt or DIC/Oxyma. The elongation of the peptide proceeded through repetitive coupling and Fmoc deprotection reactions until the desired linear precursor was achieved. Since the resin contained a target linear peptide with a Boc protective group, a solution of 5 mL of AcOH:TFE (2:2:6) was added to cleave the desired peptide. The reaction mixture was shaken for 2 h, filtered, and the filtrate was collected. The filtrate was concentrated using a rotary evaporator. The resulting crude linear peptide was characterized using HR-TOF-MS (*m/z* [M+H]^+^ 810.4730). 

### 4.3. Cyclization of Pipecolisporin Linear Peptide

The linear peptide crude was dissolved in DCM at a concentration of 1 mM. To this solution, 0.4 mmol of HATU in 0.5 mL of DMF and 1.6 mmol of DIPEA were added. The reaction mixture was stirred for 72 h at room temperature and then concentrated. Subsequently, Boc deprotection was carried out by adding a 5 mL solution of 20% TFA in DCM. The reaction mixture was shaken for 2 × 10 min, filtered, and the filtrate was collected and concentrated using a rotary evaporator. The crude product was then characterized using mass spectrometry (HR-TOF-MS). Further purification was conducted using semi-preparative RP-HPLC on the C18 column (5 mm, 10 mm × 250 mm, Phenomenex in Waters 2998, photodiode array detector, LiChrospher 100) using eluents of 100% H_2_O as solvent A and 100% acetonitrile as solvent B). The separation was performed using a linear gradient of 0% to 80% solvent B within 60 min with a flow rate of 2.0 mL min^−1^. The purified products were analyzed for purity using analytical RP-HPLC C18 column (5.0 mm, 4.6 mm × 250 mm, COSMOSIL) with solvent A of 99.9% H_2_O containing 0.1% TFA and solvent B of 99.9% acetonitrile containing 0.1% TFA with a flow rate of 1 mL min^−1^) and characterized using spectroscopic methods including HR-TOF-MS, ^1^H-NMR, and ^13^C-NMR.

### 4.4. Antimalarial Activity Assay of Pipecolisporin Peptide

Synchronized ring-stage parasite suspensions (180 µL) (2% parasitemia, 2% hematocrit) were distributed into 96-well microtiter plates, with each well containing 20 µL of varying concentrations of pipecolisporin [16,37]. Artemisinin was used as the standard drug. Untreated parasites and culture medium served as negative controls and blanks, respectively. After 48 h of incubation under standard parasite culture conditions, GelGreen^®^ (1× final concentration) was added to the wells. The plates were then wrapped in aluminum foil and incubated for an additional hour at room temperature [38]. A microplate reader was used to measure the total fluorescence of GelGreen^®^, excited at 490 nm and emitted at 530 nm, to assess parasite growth inhibition (%) at each concentration [39]. The IC_50_ values of the compounds were calculated using probit regression analysis (GraphPad Prism software, Version 8) [40].

### 4.5. Computational Analysis of Pipecolisporin Peptide

#### 4.5.1. Density Functional Theory (DFT) Methods

The Gauss View 5.0 software was employed to visualize the computational results, while the Gaussian 09 suite was utilized for executing density functional theory (DFT) calculations [41]. For the purpose of optimizing specific phytochemicals, the B3LYP hybrid functional of DFT was applied in conjunction with a 6-31G basis set. This particular combination, B3LYP/6-31G, has been previously validated in optimizing other natural compounds and is known for delivering highly accurate results.

#### 4.5.2. Molecular Docking Methods

The docking of the pipecolisporin compound was conducted using Autodock Tools version 1.5.6. The three-dimensional structures of dihydrofolate reductase (2BL9) [42], plasmepsin V (4ZL4) [43], and lactate dehydrogenase (1CET) [44] were sourced from the Protein Database (PDB) (https://www.rcsb.org/) (accessed on 18 September 2024) and converted into pdbqt format prior to docking. The proteins 2BL9, 4ZL4, and 1CET were precisely prepared by incorporating polar hydrogens, Kollman charges, and calculating fragment volumes [45]. Furthermore, specific grid boxes—2BL9 (64.00, 70.00, 60.38), 4ZL4 (70.99, 71.13, 70.93), and 1CET (60.903, 75.211, 50.674)—were defined to encompass the active sites of the respective proteins. With the aid of the auto-grid tool, grid parameter files were generated for the docking setup. Throughout the process, both the ligand and protein were treated as flexible entities. The docking results identified the most favorable binding position with the highest docking score for further analysis.

#### 4.5.3. Pharmacokinetic and Pharmacodynamics Evaluations

Bioavailability refers to the proportion of a drug dose that successfully enters the bloodstream and becomes active to produce therapeutic effects. In contrast, metabolic stability evaluates how prone a compound is to being broken down by metabolic processes, which can influence its duration in the body and its effectiveness overall. To analyze pharmacokinetic properties such as heavy atom count, predicted solubility (ESOL Log S and mol/L), gastrointestinal (GI) absorption, and blood–brain barrier (BBB) permeability of the selected phytochemicals, the Swiss ADME tool was utilized [46]. Lipinski’s rule values for the ligands were retrieved from the PubChem database [47].

#### 4.5.4. Molecular Dynamic Simulation Methods

Due to pipecolisporin’s significant interaction with malaria target proteins observed in experimental and molecular docking studies, it was chosen for detailed Molecular Dynamics (MD) simulations. The stability of the ligand–protein interaction was analyzed using MD simulations within a system containing Na+ and Cl−. The Gromacs 2016.3 software, developed by the University of Groningen, Stockholm, and the Max Planck Institute, was employed to examine the behavior of the pipecolisporin compound [48,49,50]. Simulations were carried out using the Amber ff99SB-ILDN force field to accurately capture the complex interactions between the ligand and protein [51]. For precise simulation results, the ligand–protein complex was submerged in a TIP3P water model within a cubic box, ensuring a 12 Å buffer zone, using Gromacs’ system builder tool. To neutralize the system, appropriate ions were added, and 0.15 M NaCl was included to maintain equilibrium. Following 10,000 steps of steepest descent minimization, the system was gradually heated from 0 to 300 K under NVT conditions. To optimize the environment, a 5-ns thermal relaxation was conducted, applying the Nose–Hoover Chain thermostat and Martyna–Tobias–Klein barostat methods to stabilize the pressure [52,53]. Afterward, key conditions such as particle number, heavy atom count, charge, and pressure were carefully maintained under NPT ensemble conditions to ensure stability throughout the simulation. The system was then simulated for 500 ns with a 12 Å cutoff distance, with trajectory data recorded by capturing 5000 frames at intervals of 4.8 picoseconds. The binding-free energy between the ligand and protein, along with the contributions of individual residues to the binding-free energy, was estimated using MM/PBSA on representative frames from the entire 500 ns MD simulation [54,55].

## 5. Conclusions

In conclusion, pipecolisporin was successfully synthesized using both solid-phase and solution-phase methods, yielding 30%. The compound exhibited significant antimalarial activity, consistent with computational analyses showing its effectiveness against *Plasmodium* species. MM-PBSA calculations confirmed the peptide’s stability when binding to key receptors such as dihydrofolate reductase, plasmepsin V, and lactate dehydrogenase, further supporting previous findings on the greater stability of cyclic peptides over linear ones. This research also underlined the importance of hydrophobic interactions, particularly van der Waals forces, in stabilizing these ligand–protein connections, which are crucial for membrane penetration and biological function. Additionally, considering the potential cytotoxicity observed in similar peptides, future research on pipecolisporin should focus on enhancing its therapeutic benefits while minimizing any adverse effects. These combined insights offer a robust foundation for ongoing efforts to refine pipecolisporin’s structure for improved efficacy and safety.

## Figures and Tables

**Table 1:** ^1^H- and ^13^C-NMR spectral data of synthetic pipecolisporin in CD_3_OD and comparison with its natural product.

Amino Acid	Pipecolisporin Product Synthesis (CD_3_OD, 700 MHz)		Pipecolisporin Natural Product (DMSO-d6, 500 MHz) [6]	
	^1^H δ_H_ (ppm), m, J (Hz)	^13^C (ppm)	^1^H δ_H_ (ppm), m, J (Hz)	^13^C (ppm)
**Pro** CO 1 2 3 3e 4	3.99–4.00 (m) 2.36–2.38 (m) 2.13–2.14 (m), 1.85–1.86 (m) 3.78–3.80 (m)	173.1 62.9 29.2 25.4 46.9	3.99, (m) 2.19 (m), 1.77 (m) 1.99 (m), 1.84 (m) 3.72 (m), 3.56 (m)	171.8, C 62.9, CH 29.4, CH_2_ 25.7, CH_2_ 47.3, CH_2_
**Ile** CO NH amide 1 2 3 4 2-Me	6.59, d, 8.61 4.22–4.23 (m) 2.01–2.03 (m) 1.30–1.33 (m) 0.80–0.81 (m) 0.81–0.82 (m)	172.5 57.8 36.9 24.5 10.7 14.8	6.47, d, 8.0 4.01 (m) 1.91 (m) 1.30 (m) 0.82 (m) 0.81 (m)	171.2, C 57.5, CH 37.0, CH 24.7, CH_2_ 12.1, CH_3_ 16.2, CH_3_
**β–Ala** CO NH amide 1 2	6.59, d, 8.61 2.55–2.56 (m) 3.89–3.90 (m)	171.4 34.8 34.0	7.05 (m) 2.43 (m) 3.71 (m)	171.0, C 35.2, CH_2_ 35.0, CH_3_
**Trp** CO NH amide 1 2 3 4 NH 5 6 7 8 9 10	8.47, s 4.68–4.67 (m) 3.20–3.31 (m), 3.18–3.20 (m) 7.37, d, J = 6 Hz 7.44–7.45 (m) 7.13–7.14 (m) 7.02–7.03 (m) 7.57 (d, J = 6 Hz)	171.3 51.4 27.2 108.2 123.8 136.7 118.9 121.4 111.1 118.0 127.4	8.56, d, 8.9 4.49 (m) 3.06, (d), 14.8 3.17, (dd), 14.8, 4.1 7.29, d, 2.0 11.00, (bs) 7.34, d, 8,1 7.06 (m) 6.97 (m) 7.44 (m), 8.0	170.9, C 51.8, CH 27.6, CH_2_ 108,9, C 125.0, CH 136.7, C 118.7, CH 121.7, CH 108.9, CH 118.9, CH 127.7, C
**Pipe** CO 1 2 3 4 5	3.94–3.95 (m) 1.43–1.44 (m) 0.98–0.99 (m) 1.18–1.20 (m) 3.26–3.28 (m)	171.0 56.9 20.13 19.22 23.6 39.8	3.66 (m) 1.45 (m) 0.99 (m) 1.25 (m) 3.32 (m)	169.6, C 56.7, CH 23.6, CH_2_ 20.7, CH 23.9, CH_2_ 39.4, CH_2_
**Leu** CO NH amide 1 2 3 4 4’	8.47, s 4.45–4.46 (m) 1.86–1.87 (m) 1.70–1.72 (m) 0.81–0.82 (m) 0.89–0.91 (m)	173.3 50.3 38.5 22.4 10.1 23.3	8.95, d, 8.5 4.53 (m) 1.85 (m) 1.62 (m) 0.81 (m) 0.90 (m)	173.5, C 50.3, CH 38.3, CH_2_ 25.2, CH 20.6, CH_3_ 23.9, CH_3_

**Table 2:** Binding affinity analysis of pipecolisporin with selected protein targets (2BL9, 4ZL4, 1CET).

Compound	Target Receptor	Binding Affinity	Inhibition Constant	Amino Acid Interaction	Category
Pipecolisporin	dihydrofolate reductase (2BL9)	−10.26 kcal/mol	29.90 nM (nanomolar)	A:PHE57 A:PHE57 A:LEU45 A:CYS49 A:LEU45 A:MET54 A:LEU45 A:ILE173	Hydrophobic Hydrophobic Hydrophobic Hydrophobic Hydrophobic Hydrophobic Hydrophobic Hydrophobic
	plasmepsin V (4ZL4)	−5.38 kcal/mol	113.76 uM (micromolar)	A:GLU141:O A:LYS432 A:VAL434 A:VAL434 A:VAL434 A:LEU179	Hydrogen Bond Hydrophobic Hydrophobic Hydrophobic Hydrophobic Hydrophobic
	lactate dehydrogenase (1CET)	−6.59 kcal/mol	14.81 uM (micromolar)	A:TYR85:OH A:GLU122:OE2 A:ILE54:CG2 A:ILE54:CD1 A:ILE54 A:LYS118 A:ILE119 A:LYS118 A:PHE100 A:ILE54 A:ILE119 A:ALA98 A:ILE119	Hydrogen Bond Hydrogen Bond Hydrophobic Hydrophobic Hydrophobic Hydrophobic Hydrophobic Hydrophobic Hydrophobic Hydrophobic Hydrophobic Hydrophobic Hydrophobic

**Table 3:** The binding energies and individual energy components for the simulated pipecolisporin complexes.

Compound	Target Receptor	Van der Waal Energy	Electrostatic Energy	Polar Solvation Energy	SASA Energy	Binding Energy
Pipecolisporin	dihydrofolate reductase (2BL9)	−222.699 kJ/mol	−21.963 kJ/mol	150.384 kJ/mol	−26.109 kJ/mol	−120.386 kJ/mol
	plasmepsin V (4ZL4)	−128.318 kJ/mol	−28.779 kJ/mol	85.611 kJ/mol	−16.906 kJ/mol	−88.392 kJ/mol
	lactate dehydrogenase (1CET)	−141.450 kJ/mol	−14.199 kJ/mol	78.001 kJ/mol	−16.996 kJ/mol	−94.644 kJ/mol

## Data Availability

The data are contained within the article and Supplementary Material.

## Supplementary Materials

The following supporting information can be downloaded at https://www.mdpi.com/article/10.3390/molecules30020304/s1. Figure S1: Solid-phase Peptide Synthesis of Pipecolisporin; Figure S2: Chromatogram of analytical RP-HPLC of pipecolisporin; Figure S1: HR-ToF-ESI-MS spectrum of pipecolisporin; Figure S4: ^1^H-NMR (500 MHz, CD_3_OD) spectrum of pipecolisporin; Figure S2: ^13^C-NMR (500 MHz, CD_3_OD) spectrum of pipecolisporin.
