# Supplementary material for: Synthesis, Antimalarial Activity and Molecular Dynamics Studies of Pipecolisporin: A Novel Cyclic Hexapeptide with Potent Therapeutic Potential
Source: Molecules. 2025 Jan 14;30(2):304. doi: 10.3390/molecules30020304 (PMC11767488; doi:10.3390/molecules30020304)
Supplement: Supplementary file 1 [file molecules-30-00304-s001.zip › molecules-3356636-supplementary.pdf]

SUPPLEMENTARY MATERIALS

Synthesis, Antimalarial Activity and Molecular Modelling Studies of Pipecolisporin, A Novel Cyclic Hexapeptide with Potent Therapeutic Potential

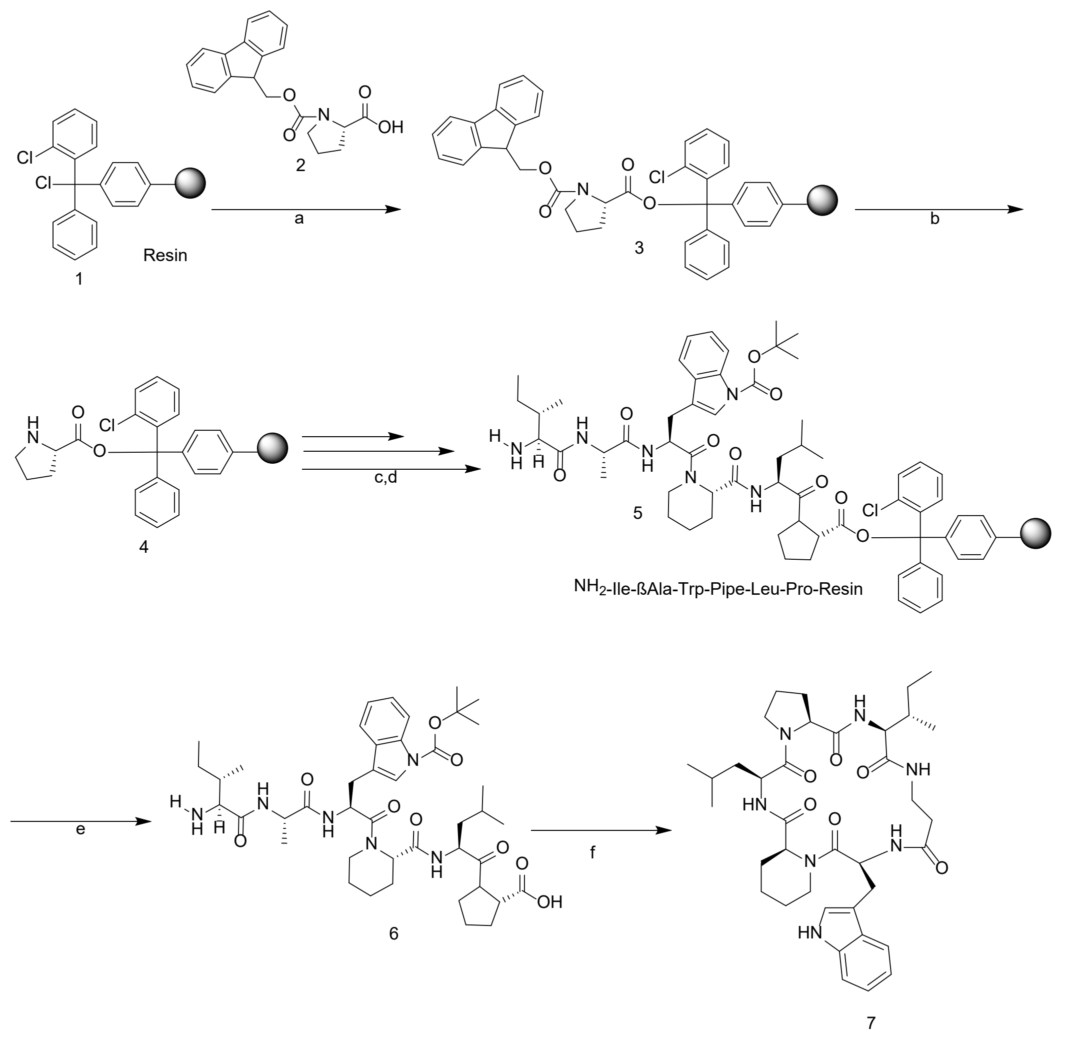

Figure S1. Solid-phase Peptide Synthesis of Pipecolisporin; (a). (1) Fmoc-L-Pro-OH (1 eq.), DIPEA (6 eq.), 4 mL CH_2Cl_2, 1h; (2) MeOH (15 eq.), DIPEA (5 eq.), CH_2Cl_2 (80 eq.), 15 min; (b) 20% piperidine in *N,N*-dimethylformamide (DMF) 10 min. (c). (1) Fmoc-L-Leu-OH, (3 eq.), HATU (3 eq.), HOAt (3 eq.), DIPEA (6 eq.), 4 mL DMF 4 hour; (2) 20% piperidine in DMF 10 min. (d). (1) Fmoc-L-Pip-OH (3 eq.), HATU (3 eq.), HOAt (3 eq.) , DIPEA (6 eq.), 4 mL DMF 4 hour; (2) 20% piperidine in DMF 10 min. (e). (1) Fmoc-L-Trp(Boc)-OH (3 eq.), HATU (3 eq.), HOAt (3 eq.) , DIPEA (6 eq.), 4 mL DMF 4 hour; (2) 20% piperidine in DMF 10 min. (f). (1) Fmoc-L-β-Ala-OH (3 eq.), HATU (3 eq.), HOAt (3 eq.), DIPEA (6 eq.), 4 mL DMF 4 hour; (2) 20% piperidine in DMF 10 min. (f). (1) Fmoc-L-Lys(Boc)-OH (3 eq.), HATU (3 eq.), HOAt (3 eq.) , DIPEA (6 eq.), 4 mL DMF 4 hour; (2) 20% piperidine in DMF 10 min. (g). (1) Fmoc-L-Ile-OH (3 eq.), HATU (3 eq.), HOAt (3 eq.) , DIPEA (6 eq.), 4 mL DMF 4 hour; (2) 20% piperidine in DMF 10 min. (h). (1) DIC (4 eq.), Oxyma (4 eq.), 4 mL DMF 72 hour. (2). TFA 2 h.

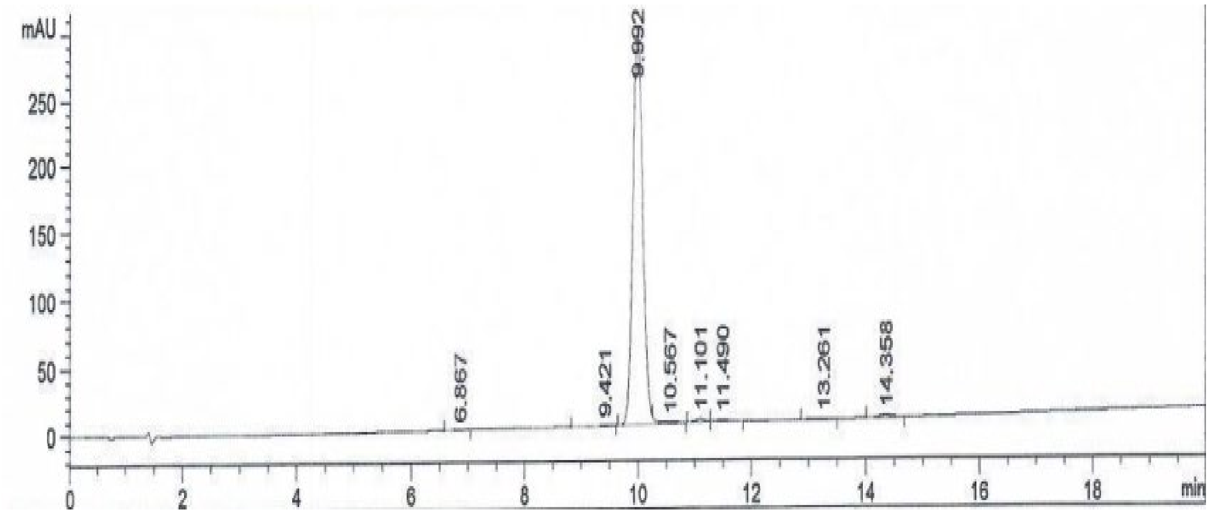

Figure S2. Chromatogram of analytical RP-HPLC of pipecolisporin

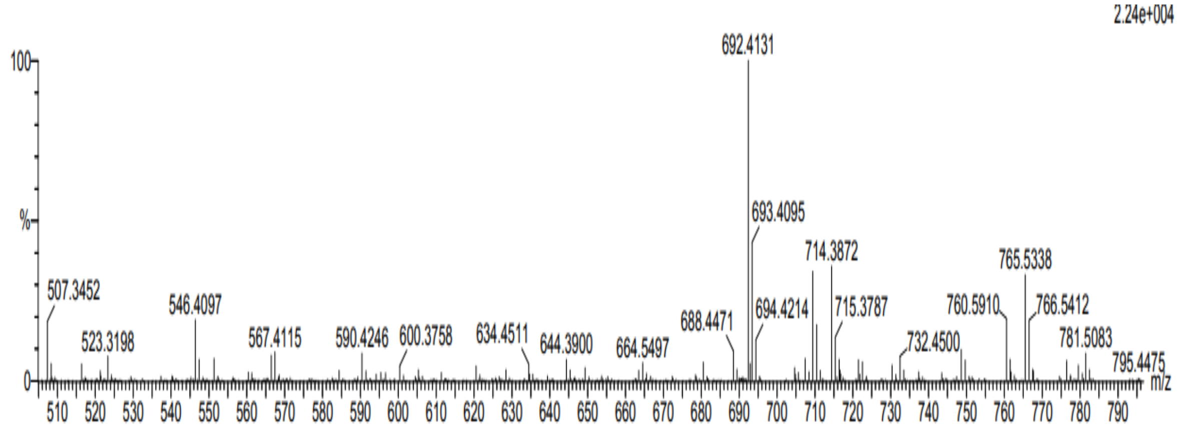

Figure S3. HR-ToF-ESI-MS spectrum of pipecolisporin

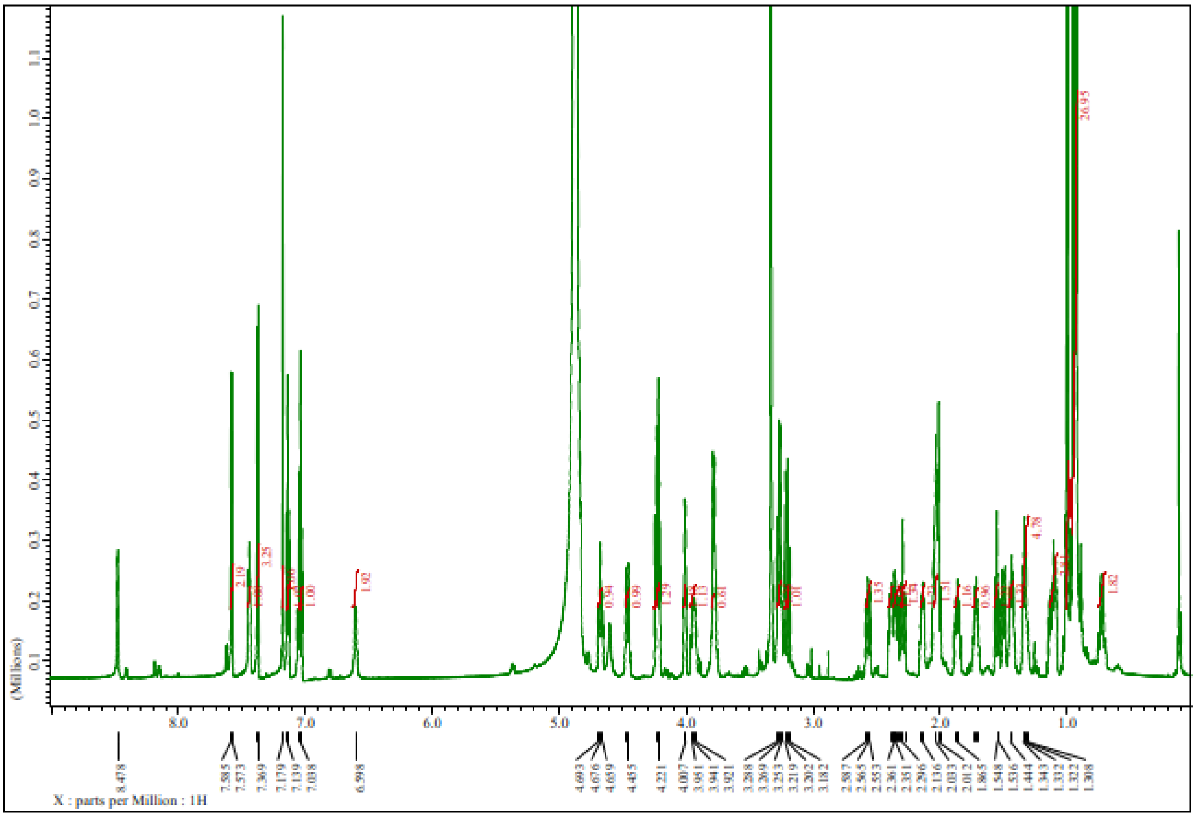

Figure S4. ^{1}H-NMR (500 MHz, CD_3OD) spectrum of pipecolisporin

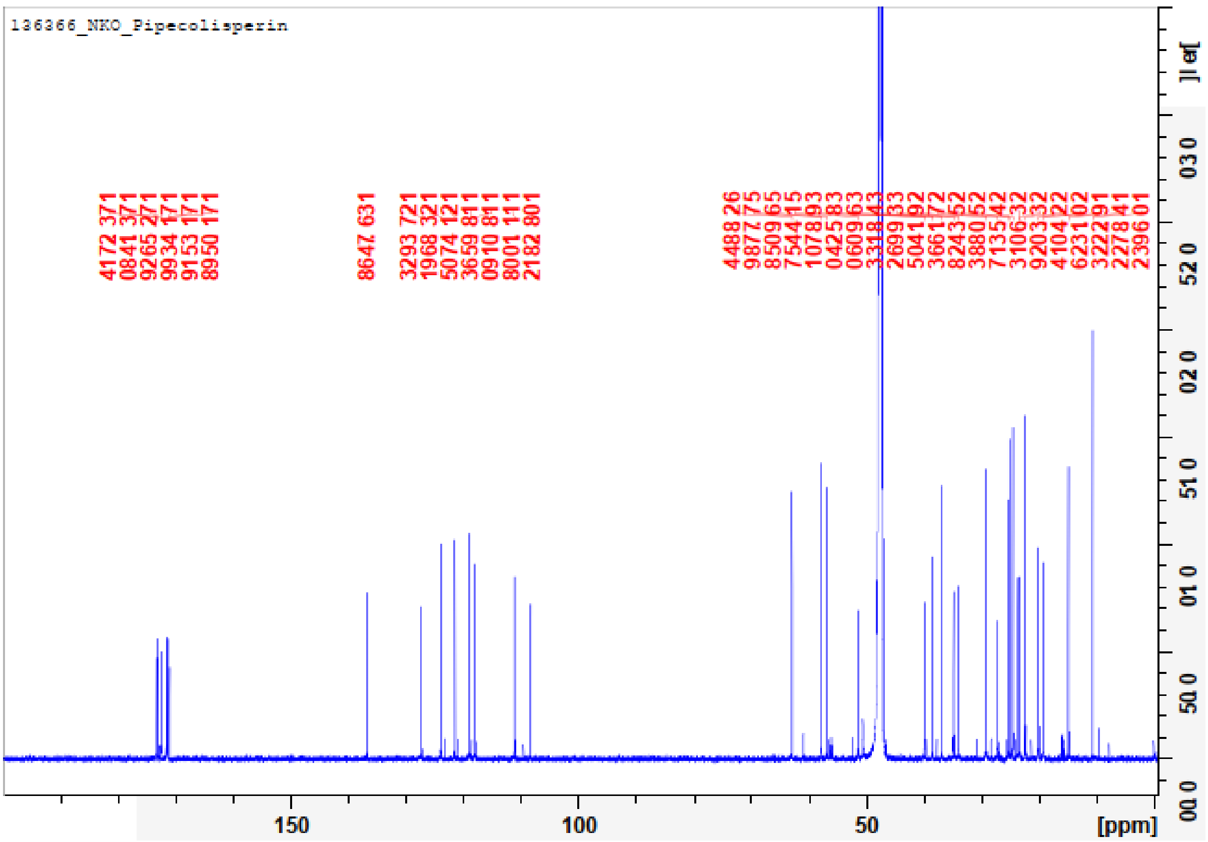

Figure S5. ^{13}C-NMR (500 MHz, CD_3OD) spectrum of pipecolisporin
